# Sero-prevalence of human brucellosis and associated factors among febrile patients attending Moyale Primary Hospital, Southern Ethiopia, 2023: Evidences from pastoralist community

**DOI:** 10.1371/journal.pntd.0012715

**Published:** 2024-12-17

**Authors:** Betrearon Sileshi, Seifu Gizaw, Belay Merkeb, Tadesse Bekele, Wuletaw Tadesse, Jafer Kezali, Endalu Tesfaye, Angefa Ayele

**Affiliations:** 1 Department of Medical Laboratory Sciences, College of Health Science, Salale University, Fiche, Ethiopia; 2 School Pharmacy, College of Health and Medical Sciences, Haramaya University, Harar, Ethiopia; 3 Ethiopian Public Health Institute, Addis Ababa, Ethiopia; 4 Department of Epidemiology, School of Public Health, Institute of Health, Bule Hora University, Bule Hora, Ethiopia; Peking University, CHINA

## Abstract

**Background:**

Brucellosis is a neglected zoonotic disease often contracted through contact with animals and consumption of unpasteurized dairy products. Despite being the most common cause of non-malarial acute febrile illness brucellosis is often misdiagnosed in healthcare setups. The global incidence of *Brucella* infection is reported to be over 2 million cases annually. In Ethiopia, there are varying reports on the prevalence of brucellosis, and just a few researches have been undertaken on the prevalence among febrile patients. Therefore, this study aimed to determine sero-prevalence of human brucellosis and associated factors among febrile patients attending Moyale Primary Hospital in southern Ethiopia.

**Methods:**

Cross-sectional study was conducted on 293 febrile patients attending Moyale Primary Hospital. We used convenience sampling technique. Blood specimen was collected and screened for *Brucella* antibody using Rose-Bengal plate test and ELISA was used for confirmation of *Brucella* infection. We used a questionnaire to collect socio-demographic data and to assess associated factors (S1 Table). Bivariate and subsequent multivariable logistic regression was performed to explore associated factors with the prevalence of human brucellosis.

**Results:**

The sero-prevalence of human brucellosis in this study was 13% (95% CI: 9.5, 16.5). Majority of the study participants (58.7%) were rural dwellers; 54.6% were male and the age range was from 5 to 80 years (mean = 24.17, SD = ±15.9). Higher number of sero-prevalence was seen among rural residents (19.8%) and male participants (14.40%). Drinking unpasteurized camel milk (AOR = 11.62, 95% CI: 3.85, 17.13; P = 0.000) and rural residence (AOR = 7.21, 95% CI: 2.48, 15.90; P = 0.000) were significantly associated with brucellosis. Consumption of pasteurized milk was shown to have protective effect (AOR = 6.12, 95% CI: 1.26, 29.76; P = 0.025) against brucellosis.

**Conclusion:**

The current study showed 13% point prevalence of human brucellosis among febrile patients attending Moyale Primary Hospital. Consuming unpasteurized milk, particularly unpasteurized camel milk, and rural residence was significantly associated with *Brucella* infection. Awareness creation about the zoonotic nature of brucellosis and the role of unpasteurized milk in the transmission of the disease is important to control human brucellosis.

## Author summary

Brucellosis is a zoonotic bacterial infection that poses significant public health challenges, particularly in pastoralist communities where human-animal interactions are frequent. The disease is often underdiagnosed due to its nonspecific symptoms, which mimic other febrile illnesses, leading to prolonged suffering and potential complications. Understanding the sero-prevalence of human brucellosis and identifying associated risk factors are crucial steps in improving diagnostic strategies and guiding public health interventions. This study focuses on febrile patients attending Moyale Primary Hospital in southern Ethiopia, a region predominantly inhabited by pastoralist communities. By examining the prevalence of brucellosis and exploring the socio-demographic and environmental factors linked to the infection, this research provides valuable insights into the burden of the disease in this vulnerable population. The findings highlight the need for targeted awareness programs, improved diagnostic protocols, and strategic public health measures to mitigate the impact of brucellosis in pastoralist settings.

## Introduction

Brucellosis is a zoonotic bacterial disease caused by several species of genus *Brucella*. The *Brucella* species are gram-negative coccobacilli, non-motile, aerobic, facultative intracellular, fastidious microorganisms [1].

Brucellosis, also known as undulant fever or Malta fever wasfirst reported in the 1850s in Malta, from a patient who was said to have acquired the infection through consumption of infected goat’s milk [2,3]. The term ‘brucellosis’ as well as the name of bacterial genus ‘*Brucella’* is named after Major-General Sir David Bruce, a military physician who in 1886 led the Malta Fever Commission that identified *Brucella melitensis* as the causative agent of the disease [4].

Brucellosis primarily affects animals, with the bacterial agent typically targeting the reproductive organs and breast tissues, leading to abortions and sterility [2,5]. Humans contract the disease through the consumption of unpasteurized dairy products, undercooked meat, or through direct contact with the bacteria via mucosal penetration [6]. Individuals handling aborted animal fetuses or uterine contents from infected animal; abattoir workers and veterinarians are at high risk of *Brucella* infection [7].

The clinical presentation of brucellosis often overlaps with acute febrile illness (AFI) caused by other pathogens, typically characterized by nonspecific flu-like symptoms manifesting as undulating fever, fatigue, sweats and malaise [8]. This similarity with other diseases can lead to misdiagnosis and under-reporting in local medical practices [9]. The course of brucellosis is usually acute, with a tendency to become chronic, leading to persistent and granulomatous disease causing recurrent fevers, arthritis, myocarditis and neuropathies [10].

Among the 12 known species of *Brucella*, *B*. *melitensis* and *B*. *abortus* are the most common ones that cause human infection followed by *B*. *suis* and *B*. *canis* [11]. Laboratory techniques for diagnosis of brucellosis include culture, serology and molecular techniques [12]. The gold standard for definitive diagnosis of brucellosis is the isolation of *Brucella* species from blood, bone marrow or other body tissues. However, the time-consuming cultivation of the bacteria as well as the risk of biological hazards, the diagnosis often relies on serological evidence of the infection [13]. The most commonly used serological tests include slide agglutination test, buffered acidified plate antigen test (BAPA), Rose-Bengal plate test (RBPT) and the enzyme linked immuno-sorbent assay (ELISA) [14].

Brucellosis constitutes a significant social and economic burden mostly affecting human populations living near animals [15]. The disease is rarely targeted by formal surveillance systems, leading to a substantial underestimation of its impact [13]. The global epidemiology of human brucellosis has drastically changed over the past years, from the previous estimate of 500,000 new cases [16] to 2.1 million annual incidence according to the Center for Disease Control (CDC) [17]. Regardless of under-reporting and the scarcity of epidemiological data, the disease is endemic in Africa [18] and its prevalence is poorly understood with varying reports from country to country [19]. In the East African Community countries, up to 44% prevalence of human brucellosis has been recorded [20].

In developing countries, including Ethiopia, little emphasis is placed on human brucellosis [21] mostly due to a lack of diagnostic services and awareness among health professionals about the disease [22]. In Ethiopia, brucellosis remains a major challenge to the development of dairy farming, affecting the livelihood of the pastoralist communities [5,23]. However, the full impact of human brucellosis is not well understood, and epidemiological data are limited [24,25].

The livelihood of the Borana community of the Moyale area mostly depends on livestock farming. In this community, bare-hand contact with aborted materials from animals and consumption of livestock products are common practices. However, the burden of human brucellosis in this area is not known. Therefore, this study aimed to determine sero-prevalence of human brucellosis and associated factors among febrile patients attending Moyale Primary Hospital in southern Ethiopia.

## Methods and materials

### Ethics statement

Ethical clearance was obtained from the Institutional Review Board of Salale University with Ref.no SLU|IRB|22|23. The study’s purpose was thoroughly explained to the data collectors and the relevant hospital authorities. Prior to specimen collection, patients were informed about the study’s objectives and written informed consent was obtained from all adult participants and the guardians of participants under the age of 12, along with assent from children aged 12 to 18. To ensure confidentiality, no personal identifiers were recorded from the charts. The research was conducted in strict adherence to the principles outlined in the World Medical Association’s Declaration of Helsinki. All tests were provided free of charge, and results were promptly communicated to the attending physician. Patients diagnosed with human brucellosis received appropriate treatment.

### Study design

Facility based cross-sectional study was conducted among febrile patients attending Moyale Primary Hospital from July to September 2023.

### Study setting

The study was conducted at Moyale Primary Hospital, located in southern Ethiopia, 770 km from the capital, Addis Ababa. The community in this region primarily depends on livestock farming and cross-border trade for their livelihood. Moyale Primary Hospital serves a population of over a million people, including residents of Moyale town, the Somali region, and neighboring cities in Kenya. The hospital offers a wide range of services, including laboratory diagnostics. The laboratory conducts various diagnostic tests for acute febrile illnesses, such as the Weil-Felix test, malaria blood film, and Salmonella rapid test.

### Study population and eligibility

The source population consisted of all febrile patients attending Moyale Primary Hospital, while the study population included all febrile patients who visited the hospital during the study period. Patients who were unable to provide consent due to critical illness or unconsciousness at the time of data collection were excluded from the study.

### Sample size and sampling procedure

The sample size of 293 was determined using a single population proportion formula, based on a brucellosis prevalence rate of 25.6% [26] reported in a study conducted among the pastoralist community of Yabello, Ethiopia. The calculation was made with a 95% confidence interval and a 5% margin of error. Participants were selected using a convenience sampling technique. To reduce sampling bias, we included individuals from various socio-demographic backgrounds and extended the sample collection period to ensure the inclusion of participants who might otherwise have been missed.

### Data collection and quality control

After reviewing relevant literature, a semi-structured questionnaire was developed. The working questionnaire was prepared in Kobocollect application. Two experienced nurses were selected for data collection at the patient site, and two laboratory professionals were chosen for biological specimen data collection. These individuals were carefully selected based on their experience and received two days of training before data collection. The questionnaire was pretested on 5% of the total sample size (15 individuals) at Moyale Health Center before the actual data collection commenced. The data collected included a wide range of potential host-related information, such as basic demographic details (age, gender, occupation, educational status, marital status, and place of residence), livestock product-related factors, and brucellosis serostatus. Quality assurance measures were implemented at every stage of the laboratory processes, with regular checks to ensure strict adherence to standard operating procedures.

### Laboratory methods

5 ml of venous blood was collected using serum separator tubes by trained medical laboratory professionals. The collected specimens were left at room temperature for 30 minutes to facilitate clotting. After the specimen had been allowed to fully clot it was centrifuged at 3000rpm for 5 minutes to get clear serum. An aliquot of the separated serum was used to screen for *Brucella* antibody using RBPT. An RBPT reagent which had performance characteristics of 89.6% specificity and 97.1% sensitivity by (Atlas Medical, Germany) was used for screening. We performed the qualitative rapid slide agglutination technique based on the manufacturer’s protocol. 50 μL of serum was dispensed on a white glossy slide and mixed thoroughly with an equal volume of RBPT reagent using a plastic applicator. The slide were then placed on a mechanical rotator and shaken for 4 minutes. Any visible agglutination was taken as a positive result. The leftover serum samples were collected in 2 ml cryotubes and stored in a freezer at negative 20-degree centigrade (-20°C). Specimens positive by the screening test were further tested by *Brucella* IgG Antibody ELISA test for confirmation. We used the BioTek 800 TS absorbance reader and Brucella IgG ELISA kit from (Tecan ltd Hamburg, Germany) for the confirmatory test. Based on the manufacturer’s instruction, diluted human serum was added to 96-well plate pre-coated with *Brucella* antigen along with positive and negative controls. This was incubated at room temperature for 1hr after which the plates were washed, conjugate then added and incubated for 30 minutes. Following a wash cycle, substrate was added and incubated for 20 minutes. The conjugate- substrate reaction was terminated by the addition of a stop solution and absorbance was read at 450nm. Finally, laboratory results were sent to the requesting physician where the questionnaire was completed by the trained data collector.

### Data management and analysis

The data was downloaded in.*csv* format and exported to SPSS version 26 for analysis. Descriptive analysis was used to summarize the socio-demographic data in the form of frequency and percentage. Since we have a dependent variable with two possible outcomes and independent variables with two or more groups, the best analytic model to explore for the association between the dependent and independent variables would be binary logistic regression. Variables with p-value (<0.25) were selected as candidate variables and included in the multivariate analysis. Before the final multivariate analysis, multi-collinearity was checked using variance inflation factor. The goodness of the model was checked using Hosmer-Lemeshow. In the multivariate analysis, variables with p-value below 0.05 were considered statistically significant.

## Result

### Socio-demographic characteristics of the study participants

The age range of the participants was from 5 to 80 years with a mean age of 24.17(SD = ±15.9) years. The majority of participants were rural residents 172 (58.7%), more than half of the participants were male 160 (54.6%) and Pastoralism was the dominant livelihood 103 (35.2%). Around one-third of the participants had no formal education 100 (34.1%) and 162 (55.3%) participants were single (**Table 1**).

**Table 1:** Demographic characteristics and Sero-prevalence of human brucellosis among acute febrile patients attending Moyale Primary Hospital, southern Ethiopia, 2023.

Factors		Total number of samples		Sero-prevalence	
		N	%	N	%
Residence	Urban	121	41.30%	4	3.30%
	Rural	172	58.70%	34	19.80%
Sex	Male	160	54.60%	23	14.40%
	Female	133	45.40%	15	11.30%
Age	<18	137	46.80%	18	13.10%
	18–35	98	33.40%	15	15.30%
	>35	58	19.80%	5	8.60%
Marital status	Single	162	55.30%	27	16.70%
	Married	116	39.60%	9	7.80%
	Divorced	6	2.00%	1	16.70%
	Widowed	9	3.10%	1	11.10%
Educational status	Illiterate	100	34.10%	16	16.00%
	Elementary	86	29.40%	7	8.10%
	High school	57	19.50%	12	21.10%
	Higher	50	17.10%	3	6.00%
Occupation	Pastoral	103	35.20%	16	15.50%
	Merchant	57	19.50%	6	10.50%
	Employee	43	14.70%	2	4.70%
	Student	90	30.70%	14	15.60%

### Sero-prevalence of human brucellosis

The sero-prevalence of *Brucella* infection among the study participants was 93/293 (31.74%) by RBPT; 38/293 (12.9%) by ELISA and the sero-prevalence is 13% (95% CI: 9.5, 16.5). The sero prevalence of human brucellosis was relatively high among rural dwellers 34/172 (19.8%) male participants 23/160 (14.4%) and in the age group of 18–35 15/98 (15.3%). The sero-prevalence was also relatively high among high school students 12/57 (21.1%) as well as among those whose marital status is single 27/162 (16.7%) (**Table 1**).

### Factors associated with Sero-prevalence of human brucellosis

Bivariate analysis was used to identify possible candidate variables for multivariate analysis and variables with p-value less than 0.25 were chosen as candidate variables. The variables "contact with aborted livestock" and "consumption of milk from aborted livestock" do not meet the requirements to be considered candidate variables because their 95% Confidence Interval of the COR includes a null value. On the other hand, the variables "marital status" and "educational status" are acceptable for the multivariate analysis since at least one group of the variables p-value was less than 0.25. Accordingly, rural residence; consumption of unpasteurized camel milk; consumption of milk from aborted livestock; contact with aborted livestock material and drinking unpasteurized milk of any livestock were all associated (**Table 2**). With these candidate variables multivariate logistic regression model was fitted to find independent predictors of human brucellosis.

**Table 2:** Factors associated with Sero-prevalence of Human Brucellosis among febrile patient attending Moyale Primary Hospital (Jul15 –Sep17), the Bivariate analysis.

Factors		Tested number	Positive	Positive (%)	COR (95%CI)	p- value
Residence	Urban	121	4	3.30%	1	0.000
	Rural	172	34	19.80%	10.54 (3.06, 36.38)	
Marital status	Single	162	27	16.70%	1	
	Married	116	9	7.80%	0.42 (0.19, 0.93)	0.033
	Divorced	6	1	16.70%	1.00 (0.11, 8.90)	1.000
	Widowed	9	1	11.10%	0.63 (0.07, 5.20)	0.66
Educational status	Illiterate	100	16	16.00%	1	
	Elementary	86	7	8.10%	2.98 (0.83, 10.77)	0.09
	High school	57	12	21.10%	1.39 (0.34, 5.63)	0.65
	Higher	50	3	6.00%	4.18 (1.11, 15.79)	0.03
Drink unpasteurized camel milk	No	188	8	4.3%	1	
	Yes	105	30	28.6%	9.00 (3.94, 20.54)	0.000
Drink raw milk from aborted livestock	No	45	2	4.4%	1	
	Yes	248	36	14.5%	3.65 (0.85, 15.74)	0.08
Drink pasteurized milk	No	205	36	17.6%	9.16 (2.15, 38.95)	0.003
	Yes	88	2	2.3%	1	
Contact with aborted livestock	No	95	7	7.4%	1	
	Yes	198	31	15.7%	2.33 (0.98, 5.51)	0.053

Finally, after controlling for potential confounding variables, the multivariate analysis for the candidate variables shows that being from rural area and consuming unpasteurized camel milk is significantly associated with human brucellosis (AOR = 7.21, 95% CI: 2.48, 15.90; P = 0.000) and (AOR = 11.62, 95% CI: 3.85, 17.13; P = 0.000) respectively. The consumption of pasteurized milk was found to have a protective effect (AOR = 6.12, 95% CI: 1.26, 29.76; P = 0.025) against *Brucella* infection (**Table 3**).

**Table 3:** Factors associated with Sero-prevalence of Human Brucellosis among febrile patient attending Moyale Primary Hospital (Jul15 –Sep17), the Multivariate analysis.

Factors		AOR (95%CI)	P-value
Residence	Urban	1	
	Rural	7.21(2.48, 15.90)	.000
Marital status	Single	1	
	Married	0.16(0.05, 0.49)	.001
	Divorced	1.77(0.16, 19.04)	.638
	Widowed	2.08(0.18, 23.61)	.555
Education	Illiterate	1	
	Elementary	0.15 (0.03, 0.83)	.029
	High school	0.04 (0.01, 0.27)	.001
	Higher	0.34 (0.47, 2.52)	.294
Drink unpasteurized Camel milk	No	1	
	Yes	11.62 (3.85, 17.13)	.000
Consume pasteurized milk	Yes	1	
	No	6.12 (1.26, 29.76)	.025

## Discussion

Human Brucellosis is a neglected zoonotic bacterial disease Predominant in developing countries. The disease has similar clinical manifestations with other febrile illnesses such as Malaria and Typhoid fever. As a result, human brucellosis is often overlooked and misdiagnosed in healthcare settings [4,9]. While the isolation of the bacteria is the gold standard for diagnosing brucellosis, the biological risks associated with bacterial culture make serology the most commonly used diagnostic method in clinical practice [14]. Despite the limited availability of epidemiological data, it is known that the disease is endemic in Africa [18]. In Ethiopia, reports on the prevalence of human brucellosis vary, ranging from 1.2% [27] to 25.6% [26] with only a few studies conducted among AFI patients. This study aimed to assess the prevalence and associated factors of this disease among AFI patients in Moyale Primary Hospital.

Of all the participants (n = 293) in this study who were presented with acute febrile illness, 13% (95% CI: 9.5, 16.5) of them had confirmed brucellosis. This result was consistent with studies done in Iraq 12.3% [28], northern Kenya 10.8% [29] and Afar, Ethiopia 15.8% [30]. However, the finding of this study was lower as compared to studies conducted in Kenya 44% [20]; Uganda 22.86% [9] and Yabello, Ethiopia 25.6% [26]. The higher prevalence from the Kenya and Ethiopia studies might be explained by the difference in the study population, where more than 88% of the study participants from the Kenyan study and the entire participants from the Ethiopian study were animal handlers, a known risk group for *Brucella* infection [31]. On the other hand, the higher prevalence in the study from Uganda could be likely due to the use of less specific laboratory test method, BAPA, and absence of a second confirmatory test.

Nevertheless, the current study reported higher sero-prevalence of human brucellosis as compared to studies in Elwayye, Ethiopia 2.7% [32], central Ethiopia 1.2% [27], Afar, Ethiopia 4.4% [33], Zanzibar 3.2% [34] and India 2.24% [35]. The possible explanation for the differences in the sero-prevalence may be due to differences in the study population, where more than 70% of the study participants from the Zanzibar study were urban dwellers, a low-risk group for *Brucella* infection. In the Indian study, it was reported that there was a good habit of consuming pasteurized milk among the majority of study participants. Thus, the lower prevalence in this study could be due to the habit of pasteurized milk consumption by the study population.

Based on the participants’ place of residence, we have noticed a higher sero-prevalence of *Brucella* infection among residents of rural areas and the multivariate analysis also shows a significant association (p<0.05) between the prevalence of human brucellosis and being from rural areas. This observation is similar to the findings reported in Kurdistan region of Iraq [28] and India [35].

A relatively higher prevalence of *Brucella* infection with no statistically significant association (p>0.05) was observed among participants aged 18–35 (15.30%) in the present study. This finding is consistent with studies conducted in Uganda [36] and Zanzibar [34]. However, in contradiction to the current study, a study conducted in Yabello, Ethiopia, showed a higher prevalence (36.4%) with significant associated (p<0.05) in the old age group [26]. Since brucellosis can occur at any age, the observed difference could be due to the smaller sample size used in the Yabello, Ethiopia study.

In this study, a slight difference in prevalence with no statistical significance (p>0.05) was observed between the two sexes, a relatively higher prevalence being in male (14.4%) than in female (11.3%) participants. The finding of our study is in line with the result of other studies conducted in Elwayye, Ethiopia [32], Yabello, Ethiopia [26], Afar, Ethiopia [30], Iraq [28], Kenya [20] and China [6]. In disagreement with the findings of the current study, a study conducted in Pakistan showed a higher prevalence in females. The difference could be due to the difference in the role of the two sexes in different communities, where in some countries females are housewives who are primarily engaged in rearing livestock and handling potentially infected products. Apart from the population difference, there is no biological or scientific basis for the sex preference of *Brucella* infection [11].

In the present study, married participants had the least prevalence (7.8%) and were negatively associated with brucellosis sero-prevalence than participants who are single (AOR = 0.157, 95%CI: 0.051, 0.489). In contrast to this finding, a higher prevalence (14.3%) of human brucellosis among divorced individuals was reported from a study conducted in Uganda [36]. Based on the higher burden of livestock handling by single men or women in the pastoral community of the Moyale area it would be reasonable to assume the role of marital status in the transmission of brucellosis. However, we could not find literature or an established scientific fact that associates marital status and human brucellosis. Thus, the difference in the two studies could be explained by the difference in the demography of the two study populations or it could be due to the difference in sample size.

In our study, high school students and participants with no formal education had the highest prevalence, (21.1%) and (16%) respectively. The multivariate analysis shows being an elementary student and being a high school student were significantly (p<0.05) and negatively associated with the prevalence of brucellosis. The finding in this study was in line with studies conducted in Elwayye, Ethiopia [32], Kenya [20] and Uganda [36]. Considering the scientifically known routes of transmission and prevention methods for *Brucella* infection, it is logical to expect an association between having no formal education and Brucellosis.

The present study has shown a strong association (p<0.05) between consumption of unpasteurized camel milk and human brucellosis (AOR = 11.62, 95% CI: 3.85, 17.13; P = 0.000). However, the finding of our study contradicts with a study conducted in Kenya [29] which reported no association between camel milk consumption and human brucellosis. This difference might be due to the low prevalence of *Brucella* infection in camels as compared to other livestock in that area or it might be due to the difference in milk consumption habits between the two populations.

It is a known fact that consumption of unpasteurized livestock milk is associated with brucellosis [6] and studies from North-Shoa, Ethiopia [19], Tunisia [18], central Ethiopia [27], northern Tanzania [37] and Uganda [36] has concluded the same. This fact is also supported by the result of the current study which showed a significant association (p<0.05) between brucellosis and drinking unpasteurized milk (AOR = 6.12, 95% CI: 1.26, 29.76; P = 0.025).

Unlike studies conducted in southern Ethiopia [32], Kenya [29], Uganda [36] and India [35], the current study has not found an association between the prevalence of human brucellosis and consuming milk from aborted animal or contact with aborted materials. The difference could be likely due to the study design, a facility-based study, where some of the study participants could not know if the milk they consume is from aborted livestock or not. Since consuming milk from aborted animal and contact with aborted materials were both candidate variables in the bivariate analysis, the difference could also be due to the relatively smaller sample size used in our study.

## Limitations

One limitation of this study is that it was facility-based. This approach may not fully capture the prevalence of brucellosis in the wider pastoralist community, as it only includes individuals who sought care at the hospital. Additionally, the use of convenience sampling may introduces the risk of sampling bias, which could affect the representativeness of the findings. While serological testing with ELISA, which has sensitivity and specificity of 98.84% and 94.13%, respectively was employed, it is less precise compared to bacterial culture. Consequently, the study may not fully reflect the true prevalence of brucellosis in the broader community. Despite these limitations, the study has provided valuable preliminary evidence of the presence of brucellosis in an area where it was previously unknown.

## Conclusion and recommendation

The sero-prevalence of human brucellosis among febrile patients of 13% cannot be ignored. The study has revealed rural residence and drinking raw milk, particularly raw camel milk were associated with *Brucella* infection. Thus, Public health interventions on the control and prevention of brucellosis such as educating animal handlers about proper hygienic practices; pasteurization of dairy products and integrated control programs with veterinary services need to be made by community health workers and other stakeholders. Public awareness of the disease and the role of raw milk in the transmission of Human Brucellosis should be integrated into health education campaigns. Clinicians should consider the possibility of brucellosis in their differential diagnosis during patient case management of AFI especially in pastoralist areas. Finally, further community-based studies designed to identify the *Brucella* species and molecular epidemiology needs to be conducted.

## Supporting information

S1 Table Data collection tool. (PDF)

S1 Dataset Raw data. (XLSX)
