# Supplementary material for: Sero-prevalence of human brucellosis and associated factors among febrile patients attending Moyale Primary Hospital, Southern Ethiopia, 2023: Evidences from pastoralist community
Source: PLoS Negl Trop Dis. 2024 Dec 17;18(12):e0012715. doi: 10.1371/journal.pntd.0012715 (PMC11651574; doi:10.1371/journal.pntd.0012715)
Supplement: S1 Table — (PDF) [file pntd.0012715.s001.pdf]

Questionnaire

Salale University, College of Medicine and Health Sciences, department of Medical Laboratory Sciences, questionnaire for study of Sero-prevalence of human brucellosis and associated factors among febrile patients visiting Moyale Primary Hospital, Southern Ethiopia, 2023

Address ________________ Age: (*yrs*) ______ Sex ___________ Id No: __________ Date: _________

	**Part 1 – Socio-demography**			
1	Where do you live?			
	A) Urban		B) Rural	
2	Marital status			
	C) Single	D) Married	E) Divorced	F) Widowed
3	Educational status			
	A) Illiterate	B) Elementary	C) High school	D) Higher
4	Occupational status			
	A) Pastoral	B) Merchant	C) Employee	D) Other
5	Family income how much is your family income per month in birr?			
	A) <1000	B) 1000-3000	C) 3001-5000	D) >5000
6	Family size			
		A) 1-5	B) 6-10	C) 10+
7	Do you have cattle or small ruminant at your home?			
	A) Yes	B) No		
8	If yes for Q#7, which animal do you have? *(more than one answer is possible)*			
	A) Goat	B) Sheep	C) Cattle	D) Camel
	**Part 2 – Associated factors**			
9	Do you consume milk?			

	A) Yes	B) No		
10	If yes for Q#9 milk of which animal do you consume? *(more than one answer is possible)*			
	A) Goat	B) Sheep	C) Cattle	D) Camel
11	If yes for Q#9 do you boil/pasteurize milk?			
	A) Yes	B) No		
12	Do you consume yoghurt?			
	A) Yes	B) No		
13	Do you consume raw meat?			
	A) Yes	B) No		
14	Do you consume fresh animal blood?			
	A) Yes	B) No		
15	Do you drink raw milk from aborted animals?			
	A) Yes	B) No		
16	Have ever touched aborted fetus/ uterine discharge with your bare hand?			
	A) Yes	B) No		
	**Part 3 – Clinical data**			
17	Body temperature? ________________________			
18	Grade of fever?			
	A) Low	B) Moderate	C) High	D) Hyperpyrexia
19	Onset of febrile illness? ____________________ (*days*)			
20	Clinical symptoms?			
	A) Weakness	B) Vomiting	C) Headache	D) Malaise
	E) Joint pain	F) Others, specify ________________		
21	RBPT result?			
	A) Negative	B) Positive		
22	ELISA result?			
	A) Negative	B) Positive		
